# Optical Coherence Tomography Angiography of Peripapillary Vessel Density in Multiple Sclerosis and Neuromyelitis Optica Spectrum Disorder: A Comparative Study

**DOI:** 10.3390/jcm10040609

**Published:** 2021-02-05

**Authors:** Małgorzata Rogaczewska, Sławomir Michalak, Marcin Stopa

**Affiliations:** 1 Department of Ophthalmology, Chair of Ophthalmology and Optometry, Poznan University of Medical Sciences, 16/18 Grunwaldzka Street, 60-780 Poznan, Poland; malgorzata.rogaczewska@gmail.com; 2 Department of Neurochemistry and Neuropathology, Chair of Neurology, Poznan University of Medical Sciences, 49 Przybyszewskiego Street, 60-355 Poznan, Poland; swami@ump.edu.pl

**Keywords:** multiple sclerosis, neuromyelitis optica spectrum disorder, optical coherence tomography angiography

## Abstract

Multiple sclerosis (MS) and neuromyelitis optica spectrum disorder (NMOSD) are demyelinating diseases of the central nervous system, which differ in the pathogenic mechanism. A common clinical presentation of both conditions is optic neuritis (ON). The study aimed to compare the radial peripapillary capillary (RPC) vessel density in MS and NMOSD patients using optical coherence tomography angiography (OCTA). A total of 40 MS patients, 13 NMOSD patients, and 20 controls were included. The average RPC vessel density was significantly lower in ON eyes (MS+ON, NMOSD+ON) than in non-ON eyes (MS−ON, NMOSD−ON) and in MS+ON, MS−ON, NMOSD+ON, and NMOSD−ON compared with the control group. In NMOSD+ON eyes, the vessel density in superior nasal, nasal superior, and inferior sectors was significantly more decreased than in MS+ON eyes. RPC reduction was also observed in inferior nasal and temporal superior sectors in MS−ON eyes compared with NMOSD−ON eyes. In conclusion, our findings indicate that optic neuritis is associated with a more significant RPC vessel density drop in NMOSD than in MS patients, and the predilection to superior and inferior sectors may be useful as a differential diagnostic marker.

## 1. Introduction

Multiple sclerosis (MS) and neuromyelitis optica spectrum disorder (NMOSD) are central nervous system (CNS) diseases characterized by inflammation, demyelination, and axonal loss [1,2]. Unlike in MS, a serum autoantibody marker can be found in up to 80% of NMOSD patients [3]. This highly specific immunoglobulin G (IgG) targets the protein aquaporin-4 (AQP4), a water channel presented in the membranes of astrocytes in the CNS. Within the eye, the astrocytes are mainly restricted to the retinal nerve fiber layer (RNFL), consisting of ganglion cells axons [4]. Optic neuritis (ON), a common manifestation of both diseases, is often bilateral and has a more severe course with poorer visual prognosis in NMOSD patients [5,6]. Moreover, the thinner RNFL after ON in NMOSD eyes suggests a more widespread axonal damage [7,8].

The RNFL is supplied by a radial peripapillary capillary (RPC) plexus that runs parallel with the axons. By using optical coherence tomography angiography (OCTA), a non-invasive in vivo imaging technique, the RPC can be visualized. The OCTA software also provides automatic vessel segmentation, and evaluation presented quantitatively as a vessel density (VD) parameter [9].

RPC’s vessel density was independently evaluated in MS and NMOSD patients in several studies [10,11,12,13,14]. Spain et al. reported that in MS patients, the RPC density was significantly reduced in ON (MS+ON) and non-ON (MS−ON) eyes compared with controls, but in the study of Wang et al., the RPC of MS−ON eyes did not differ from healthy eyes. However, both studies showed that the MS+ON group had lower RPC than the MS−ON group [11,12]. Regarding the NMOSD patients, the studies were consistent in their findings that the peripapillary vessel density was significantly decreased in NMOSD+ON and NMOSD−ON groups compared with controls and in NMOSD+ON compared with NMOSD−ON [13,14]. However, a comparative analysis of RPC in MS and NMOSD eyes has not been reported so far.

The aim of this study was to investigate and compare the peripapillary vessel density in MS and NMOSD patients in regard to the history of optic neuritis.

## 2. Materials and Methods

### 2.1. Ethical Approval

The study was approved by the medical ethics committee of the Poznan University of Medical Sciences (approval No. 562/18 from May 2018) and was performed in accordance with the Declaration of Helsinki. Written informed consent was obtained from all subjects after an explanation of the nature of the study.

### 2.2. Study Participants

In this observational study, we recruited relapsing-remitting MS and AQP4-IgG seropositive NMOSD patients at the Department of Ophthalmology and the Department of Neurology of the Poznan University of Medical Sciences from June 2018 to September 2020. All patients with MS fulfilled the revised 2017 McDonald criteria [15]. NMOSD patients were diagnosed according to the revised 2015 NMOSD diagnostic criteria [16]. The anti-aquaporin-4 antibodies were detected by means of indirect fluorescence using a commercial cell-based assay with aquaporin 4 transfected cells (EUROIMMUN AG, Lübeck, Germany). Analyses were performed in the Department of Neurochemistry and Neuropathology at the Poznan University of Medical Sciences, which participates in an international external quality control system and receives regular certification for the detection of AQP4-IgG (Institut für Qualitätssicherung, Lübeck, Germany). Clinical data, including disease duration and the number of ON attacks, were recorded. We divided the patients’ eyes into subgroups: eyes with prior optic neuritis (MS+ON, NMOSD+ON) and eyes with no history of ON (MS−ON, NMOSD−ON). Age- and sex-matched healthy volunteers served as controls.

All participants underwent an ocular examination, which included best-corrected visual acuity (BCVA) measurement, Goldmann applanation tonometry with central corneal thickness correction, slit-lamp biomicroscopy, indirect ophthalmoscopy, spectral-domain OCT (SD-OCT), and OCT angiography. Visual acuity was assessed with The Early Treatment of Diabetic Retinopathy Study chart and converted to logMAR notation.

The study inclusion criteria were age ≥ 18 years, no ON attack within 6 months prior to the examination, and at least 2 years of disease duration for MS patients. The participants with myopia greater than 6 diopters, optic disc drusen, hypertensive or diabetic retinopathy, glaucoma, history of uveitis, or eye surgery were excluded from the study. The eyes with low OCT image quality were not incorporated into the analysis.

### 2.3. SD-OCT

The peripapillary retinal nerve fiber layer thickness was obtained using the optic nerve head protocol and measured around a 3.45 mm diameter circle centered on the optic disc (RTVue XR Avanti with AngioVue, Optovue Inc., Fremont, CA, USA; software version 2017.1.0.151). The RNFL thickness of the average total and the automatically generated 8 sectors, i.e., superior temporal (ST), superior nasal (SN), nasal superior (NS), nasal inferior (NI), inferior nasal (IN), inferior temporal (IT), temporal inferior (TI), and temporal superior (TS) were analyzed.

### 2.4. OCT Angiography

OCTA is a non-invasive and non-dye-based imaging modality based on a split-spectrum amplitude-decorrelation angiography algorithm. It detects the blood motion in the vessels through sequentially obtained OCT cross-sectional scans. The flow density maps show the perfused retinal blood vessels and provide the quantitative percentage information of the vascularized area expressed as vessel density [17,18,19].

The OCTA en face image acquisition was performed with RTVue XR Avanti with AngioVue (Optovue Inc., Fremont, CA, USA; software version 2017.1.0.151). The radial peripapillary capillary plexus was visualized using a 4.5 × 4.5 mm rectangle scan centered on the optic nerve head, and the peripapillary area was defined as a 1.0 mm wide round annulus extending outward from the optic disc boundary. The AngioVue software automatically analyzed the vessel density of the superficial retinal layers extended from the internal limiting membrane to the outer boundary of the nerve fiber layer. We investigated the average vessel density of the peripapillary area and the 8 sectors, as mentioned earlier.

The low-quality images with the signal strength index < 50 or significant motion artifacts were excluded from the analysis.

### 2.5. Statistical Analysis

Statistical analysis was performed using Statistica v13.1 (StatSoft, Inc., Tulsa, OK, USA) and SPSS (SPSS, Inc., Chicago, IL, USA). Data were tested by the Shapiro–Wilk test to determine the normality of continuous variables. Differences among the cohorts were tested using the Chi-square test for sex and the Kruskal–Wallis test for age and BCVA. The Mann–Whitney *U*-test was used to evaluate differences in the time since the last ON attack and disease duration between groups. To account for intrasubject inter-eye dependencies, we used generalized estimating equation models for comparison of SD-OCT and OCTA parameters between cohorts. Correlations between RPC vessel density and RNFL thickness were assessed with Pearson’s *r* correlation test. Due to the exploratory nature of this study, no adjustment for multiple comparisons was performed. Statistical significance was established at *p* < 0.05.

## 3. Results

### 3.1. Study Population

A total of 40 MS patients, 13 NMOSD patients, and 20 healthy controls were enrolled. Five eyes of MS and six eyes of NMOSD patients were excluded from analysis due to the low image quality of the OCTA scan. The median time since the last ON attack (in years) and interquartile range were 6.5 (4–8) for MS and 9.0 (4–9) for NMOSD patients (*p* = 0.629). No significant differences were observed between patients and controls on age, sex, and disease duration, except for BCVA (*p* < 0.001). Demographic and clinical features are detailed in Table 1.

### 3.2. SD-OCT

**Figure 1:**
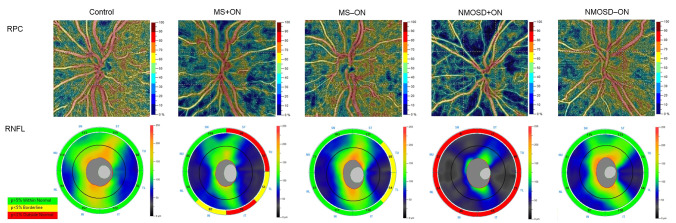
Representative OCT angiography and spectral-domain OCT images of the healthy, MS+ON, MS−ON, NMOSD+ON, and NMOSD−ON left eyes. The color-coded flow density maps present reduced retinal peripapillary vessel density in eyes with a history of optic neuritis, more pronounced in the NMOSD+ON eye. The attenuation of large retinal vessels is only seen in the NMOSD+ON eye. The corresponding RNFL thickness maps show an axonal loss on the temporal side of the optic disc in the MS+ON and MS−ON eyes, whereas, in the NMOSD+ON eye, all sectors were markedly thinner. MS = multiple sclerosis; NMOSD = neuromyelitis optica spectrum disorder; OCT = optical coherence tomography; ON = optic neuritis; RNFL = retinal nerve fiber layer; RPC = radial peripapillary capillaries.

**Figure 2:**
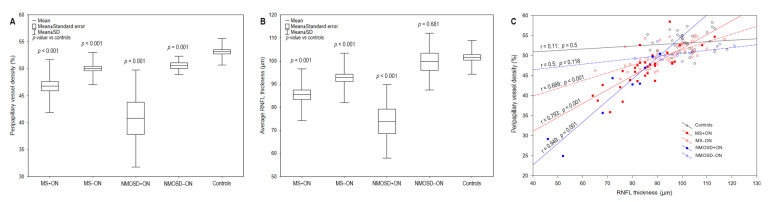
The boxplots demonstrate the average peripapillary vessel density (**A**) and RNFL thickness (**B**) of MS+ON, MS−ON, NMOSD+ON, NMOSD−ON, and controls. The scatterplot presents the correlation between RPC and RNFL parameters of all groups (**C**). MS =multiple sclerosis; NMOSD = neuromyelitis optica spectrum disorder; ON = optic neuritis; RNFL = retinal nerve fiber layer; RPC = radial peripapillary capillaries; SD = standard deviation.

**Figure 3:**
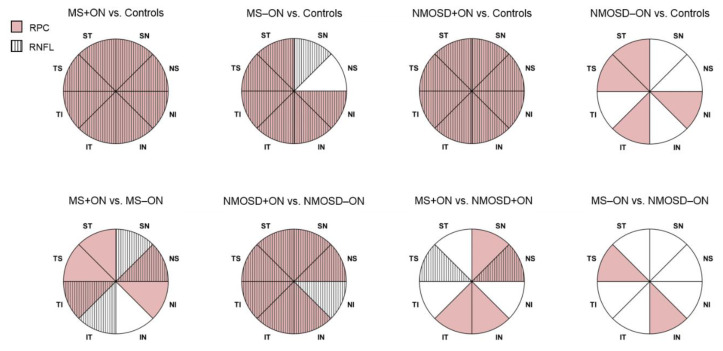
Comparison of the sectoral RPC vessel density and RNFL thickness. The color- and pattern-coded sectors illustrate the significant differences (*p* < 0.05) of the corresponding RPC (pink) and RNFL (lines) between patients and controls (top row) and between selected groups (bottom row). The exact *p*-values are presented in Table 3 and Table 4. IN = inferior nasal; IT = inferior temporal; MS = multiple sclerosis; NI = nasal inferior; NMOSD = neuromyelitis optica spectrum disorder; NS = nasal superior; ON = optic neuritis; RNFL = retinal nerve fiber layer; RPC = radial peripapillary capillaries; SN = superior nasal; ST = superior temporal; TI = temporal inferior; TS = temporal superior.

The average RNFL thickness was lower in MS+ON, MS−ON, and NMOSD+ON groups than in controls (*p* < 0.001; Table 2 and Table 3, Figure 1 and Figure 2B). The detailed analysis revealed that compared with healthy eyes, the RNFL was significantly thinner in all sectors in MS+ON and NMOSD+ON eyes and in all sectors except for NS in MS−ON eyes (Table 2 and Table 3, Figure 3). In patients with the same diagnosis, the average RNFL reduction was seen in ON eyes compared with non-ON eyes (*p* = 0.007 for MS, *p* < 0.001 for NMOSD; Table 3). The SN, NS, IT, and TI sectors were the only ones that differed in axonal loss between MS+ON and MS−ON, whereas in NMOSD groups, the RNFL in ON eyes was more affected in all sectors (Table 3, Figure 3). In NMOSD+ON eyes, the axonal loss was only noted in NS (*p* = 0.004) and TS (*p* = 0.017), compared with MS+ON eyes. However, the average and sectoral RNFL thickness were comparable between MS−ON and NMOSD−ON groups (Table 3, Figure 3).

### 3.3. OCTA

In MS+ON, MS−ON, NMOSD+ON, and NMOSD−ON groups, the average RPC vessel density was significantly decreased compared with the control group (Table 2 and Table 4, Figure 1 and Figure 2A). Regarding the peripapillary sectors, the vessel density in MS+ON and NMOSD+ON was significantly reduced in all sectors, in MS−ON—in all sectors except for SN and NS ones, and in four sectors (ST, NI, IT, TS) in NMOSD−ON, compared with controls (Table 2 and Table 4, Figure 3). The average peripapillary vessel density in MS and NMOSD patients was also lower in ON eyes than in non-ON eyes (*p* < 0.001 and *p* = 0.003, respectively; Table 4). The RPC in temporal, nasal and ST sectors were decreased in MS+ON compared with MS−ON (Table 4, Figure 3). In NMOSD groups, the vessel density in ON eyes was significantly reduced in all sectors except for NI (Table 4, Figure 3). Moreover, in NMOSD+ON eyes, the sectoral vessel density was significantly lower in both inferior, SN and NS sectors than in MS+ON, and the MS−ON eyes had lower RPC density in IN and TS than NMOSD−ON (Table 4, Figure 3).

### 3.4. Association of OCTA and SD-OCT

A strong positive correlation was found between radial peripapillary vessel density and RNFL thickness in MS+ON (r = 0.793, *p* < 0.001), MS−ON (r = 0.689, *p* < 0.001), and NMOSD+ON (r = 0.949, *p* < 0.001) groups (Figure 2C).

## 4. Discussion

This study is the first to compare the radial peripapillary vessel density alterations in MS and NMOSD patients using OCTA. We found that all RPC and RNFL sectors were lower in MS and NMOSD eyes after optic neuritis than in healthy eyes. Comparing MS+ON and NMOSD+ON groups, the vessel density was significantly reduced in NMOSD+ON eyes with a predilection to inferior, superior nasal, and nasal superior sectors. This involvement pattern might be related to the large retinal vessels’ location from the superior and inferior edge of the optic disc. As presented in Figure 1, these vessels were significantly attenuated in an exemplary NMOSD+ON eye.

Green and Cree reported that the narrowing of the peripapillary vascular tree observed through fundoscopy was a feature of NMOSD eyes [20]. They also suggested that the vessel attenuation in NMOSD+ON was not secondary to the reduced metabolic demand of atrophied retina because, in MS+ON eyes, the large vessels had normal appearance even with markedly reduced RNFL [20]. Although the cause of the retinal vessels’ alteration in NMOSD+ON is not entirely elucidated, the explanation may be sought in a distinct immunopathological mechanism. The retinal peripapillary vessels are enveloped by the end-foot processes of macroglial cells, i.e., astrocytes and Müller cells, which form and maintain the inner blood–retina barrier. The perivascular end-feet are enriched with aquaporin-4, a water channel which is targeted by AQP4-IgG in NMOSD under inflammatory conditions [21]. The pathological studies of NMO lesions revealed that the blood vessels had a narrow lumen and thickened, hyalinized or fibrotic walls [22,23]. However, while similar studies of retinal tissue were lacking, it might be speculated that the attenuated peripapillary vessels had undergone the same changes.

We also observed more substantial axonal loss in NMOSD+ON than in MS+ON eyes, but only NS and TS sectors differed significantly. Several studies reported that after ON, superior and inferior portions of RNFL were thinner in NMOSD than MS eyes [8,24,25]. Interestingly, this sector-specific predilection of axonal damage was reflected in the RPC reduction in our study, indicating that the blood vessels might be more affected than axons in NMOSD+ON patients.

Comparing ON and non-ON eyes, the reduction of RPC vessel density had temporal and nasal predilection in MS, whereas in NMOSD, 7 out of 8 sectors were significantly affected. The axonal loss was observed in superonasal and inferotemporal quadrants in MS, and in all sectors in NMOSD. These results pointed out that ON led to more widespread vascular and axonal damage in NMOSD eyes. It was shown that depending on location in the retina, the ganglion cell axons varied in diameter size. Namely, the smallest-diameter axons were more abundant in the temporal sector, while inferior, superior, and nasal axons were larger [26]. Evangelou et al. demonstrated that these small parvocellular axons, mainly located in the papillomacular bundle, were preferentially susceptible to damage in MS patients [27]. Besides, in the absence of ON, the axonal loss might occur due to the retrograde trans-synaptic degeneration from MS lesions in the posterior visual pathway [28]. In our MS−ON group, the peripapillary nerve fibers were significantly thinner in 7 out of 8 sectors with parallel RPC reduction. On the contrary, in the MS+ON compared with MS−ON, the capillary density reduction was not strictly accompanied by the sectoral RNFL thinning. However, we could not unequivocally explain this phenomenon.

In MS−ON eyes, the average global vessel density and axonal layer thickness were significantly lower, whereas in NMOSD−ON eyes, only the vessel density was reduced compared with controls. Our findings were in agreement with Spain et al.’s study of MS patients, in which vascular and axonal decrease occurred independently of ON episode [11]. Regarding NMOSD, only RPC vessel density was significantly lower in non-ON eyes, as Huang et al. and Chen et al. reported [13,14]. This result was consistent with our observations.

Our study’s most clinically valuable outcome was demonstrating the difference in RPC vessel density between MS+ON and NMOSD+ON eyes. After optic neuritis, the indirect fundus examination may show retinal peripapillary vessels’ attenuation indicating the diagnosis of NMOSD, but such an assessment is very subjective. OCT angiography, which provides automatic, precise, and repeatable analysis, is a useful tool for detecting and monitoring the vessels alterations in time. We showed that the superior nasal, nasal superior, and inferior sectors of RPC vessel density were more reduced in NMOSD+ON than MS+ON eyes. Thus, we believe that the OCTA may contribute to an accurate diagnosis of demyelinating optic neuritis in disputable cases.

A limitation of our study is a small group of NMOSD patients due to the low prevalence of this disease. Some patients were also unable to complete ophthalmic examination because of visual and physical disability. Therefore, further studies with larger cohorts are required to strengthen our observations.

In conclusion, we demonstrated a distinct pattern of RPC density and RNFL thickness alterations in MS and NMOSD patients. After ON, the peripapillary vessel density was more decreased in NMOSD than MS eyes, with a predilection to superior and inferior sectors. In NMOSD, the ON was also associated with widespread vascular and axonal damage, whereas in MS patients, the reduction of vessel density and nerve fiber layer occurred independently of ON. Our findings suggest that the optic nerve inflammation may affect the peripapillary capillary plexus more in NMOSD than in MS patients.

## Figures and Tables

**Table 1:** Demographic and clinical characteristics of MS, NMOSD patients, and controls.

	MS	NMOSD	Controls
Number of subjects	40	13	20
Number of eyes enrolled ON eyes Non-ON eyes	75 30 45	20 9 11	40 - 40
Age (years), mean ± SD	35.15 ± 7.47	42.08 ± 10.23	37.90 ± 11.47
Sex (female/male)	32/8	11/2	17/3
Disease duration (years), median (min–max)	8 (3–32)	9 (1–33)	-
BCVA of enrolled eyes (logMAR), median (min–max)	0.00 (0.00–0.20)	0.00 (0.00–2.30)	0.00 (0.00–0.00)

BCVA = best-corrected visual acuity; logMAR = the logarithm of the minimum angle of resolution; max = maximum; min = minimum; MS = multiple sclerosis; NMOSD = neuromyelitis optica spectrum disorder; ON = optic neuritis; SD = standard deviation.

**Table 2:** Baseline spectral-domain OCT and OCT angiography results of patients and controls.

	MS+ON Mean ± SD	MS−ON Mean ± SD	NMOSD+ON Mean ± SD	NMOSD−ON Mean ± SD	Controls Mean ± SD
**RNFL (μm)**					
average	85.57 ± 11.39	91.00 ± 10.48	73.89 ± 15.93	99.73 ± 12.26	101.60 ± 7.46
ST	119.17 ± 15.78	125.98 ± 16.85	101.78 ± 24.30	134.64 ± 19.09	137.90 ± 13.81
SN	93.43 ± 15.75	100.18 ± 15.95	79.00 ± 23.90	111.00 ± 18.73	108.93 ± 13.36
NS	72.33 ± 10.49	79.89 ± 14.43	59.67 ± 11.98	81.55 ± 12.64	84.83 ± 14.26
NI	66.27 ± 12.35	70.16 ± 10.99	59.00 ± 13.93	77.45 ± 15.01	75.50 ± 9.19
IN	100.97 ± 17.83	104.29 ± 13.14	89.11 ± 28.53	116.64 ± 20.22	116.60 ± 14.14
IT	115.77 ± 21.78	126.87 ± 17.91	102.33 ± 24.35	133.18 ± 20.03	138.38 ± 12.73
TI	51.23 ± 12.34	58.98 ± 10.58	46.33 ± 10.31	63.00 ± 8.93	68.05 ± 8.28
TS	65.50 ± 18.23	70.96 ± 13.19	55.33 ± 12.43	79.82 ± 12.49	83.18 ± 8.84
**RPC (%)**					
average	46.76 ± 4.96	49.87 ± 3.10	40.80 ± 8.99	50.60 ± 1.71	52.97 ± 2.51
ST	50.85 ± 7.44	54.56 ± 4.53	43.53 ± 11.62	53.68 ± 2.73	57.40 ± 2.59
SN	46.52 ± 6.60	48.78 ± 5.02	36.81 ± 11.10	49.19 ± 3.02	49.98 ± 3.98
NS	45.64 ± 5.59	48.30 ± 4.05	38.79 ± 8.76	47.69 ± 2.58	49.23 ± 3.78
NI	43.41 ± 6.27	46.50 ± 4.17	40.61 ± 7.90	45.63 ± 4.08	49.09 ± 3.45
IN	48.24 ± 6.15	48.98 ± 4.22	39.09 ± 112.00	51.14 ± 2.90	52.80 ± 4.40
IT	54.16 ± 6.50	56.14 ± 4.40	43.46 ± 12.45	56.26 ± 3.33	59.99 ± 3.39
TI	41.99 ± 6.85	46.82 ± 4.85	41.56 ± 6.93	50.20 ± 4.93	52.59 ± 3.97
TS	46.14 ± 7.35	51.44 ± 4.31	43.76 ± 7.33	53.68 ± 3.67	56.10 ± 3.63

IN = inferior nasal; IT = inferior temporal; MS = multiple sclerosis; NI = nasal inferior; NMOSD = neuromyelitis optica spectrum disorder; NS = nasal superior; OCT = optical coherence tomography; ON = optic neuritis; RNFL = retinal nerve fiber layer; RPC = radial peripapillary capillaries; SD = standard deviation; SN = superior nasal; ST = superior temporal; TI = temporal inferior; TS = temporal superior.

**Table 3:** Differences in RNFL thickness among patients and controls.

	MS+ON vs. Controls		MS−ON vs. Controls		NMOSD+ON vs. Controls		NMOSD−ON vs. Controls	
RNFL (μm)	β (SE)	*p*-Value	β (SE)	*p*-Value	β (SE)	*p*-Value	β (SE)	*p*-Value
average	−16.033 (2.665)	<0.001	−9.400 (2.446)	<0.001	−27.711 (5.779)	<0.001	−1.873 (4.555)	0.681
ST	−18.733 (4.089)	<0.001	−11.922 (3.980)	0.003	−36.122 (9.103)	<0.001	−3.264 (6.714)	0.627
SN	−15.492 (4.120)	<0.001	−8.747 (3.708)	0.018	−29.925 (8.244)	<0.001	2.075 (6.690)	0.756
NS	−12.492 (3.669)	<0.001	−4.936 (3.894)	0.205	−25.158 (4.889)	<0.001	−3.280 (5.447)	0.547
NI	−9.233 (2.998)	0.002	−5.344 (2.637)	0.043	−16.500 (5.520)	0.003	1.955 (5.641)	0.729
IN	−15.633 (4.494)	<0.001	−12.311 (3.723)	<0.001	−27.489 (11.277)	0.015	0.036 (8.288)	0.996
IT	−22.608 (5.000)	<0.001	−11.508 (4.111)	0.005	−36.042 (9.291)	<0.001	−5.193 (7.943)	0.513
TI	−16.817 (2.935)	<0.001	−9.072 (2.482)	<0.001	−21.717 (3.461)	<0.001	−5.050 (3.405)	0.138
TS	−17.675 (4.184)	<0.001	−12.219 (2.931)	<0.001	−27.842 (2.632)	<0.001	−3.357 (4.436)	0.449
	**MS+ON vs. MS−** **ON**		**NMOSD+ON vs. NMOSD−** **ON**		**NMOSD+ON vs. MS+ON**		**NMOSD−** **ON vs. MS−** **ON**	
**RNFL (μm)**	**β (SE)**	***p*** **-** **Value**	**β (SE)**	***p*** **-** **Value**	**β (SE)**	***p*** **-** **Value**	**β (SE)**	***p*** **-** **Value**
average	−6.633 (2.460)	0.007	−25.838 (7.069)	<0.001	−11.678 (5.948)	0.050	7.527 (4.649)	0.105
ST	−6.811 (3.641)	0.061	−32.859 (10.421)	0.002	−17.389 (9.207)	0.059	8.659 (6.791)	0.202
SN	−6.744 (3.279)	0.040	−32.000 (9.711)	<0.001	−14.433 (8.402)	0.086	10.822 (6.646)	0.103
NS	−7.556 (2.925)	0.010	−21.879 (6.099)	<0.001	−12.667 (4.363)	0.004	1.657 (5.148)	0.748
NI	−3.889 (2.788)	0.163	−18.455 (7.410)	0.013	−7.267 (5.661)	0.199	7.299 (5.600)	0.192
IN	−3.322 (3.748)	0.375	−27.525 (13.220)	0.037	−11.856 (11.398)	0.298	12.347 (8.069)	0.126
IT	−11.100 (4.832)	0.022	−30.848 (11.551)	0.008	−13.433 (9.911)	0.175	6.315 (8.180)	0.440
TI	−7.744 (2.728)	0.005	−16.667 (4.563)	<0.001	−4.900 (3.915)	0.211	4.022 (3.534)	0.255
TS	−5.456 (4.108)	0.184	−24.485 (4.613)	<0.001	−10.167 (4.248)	0.017	8.863 (4.678)	0.058

β = regression coefficient; IN = inferior nasal; IT = inferior temporal; MS = multiple sclerosis; NI = nasal inferior; NMOSD = neuromyelitis optica spectrum disorder; NS = nasal superior; ON = optic neuritis; RNFL = retinal nerve fiber layer; SE = standard error; SN = superior nasal; ST = superior temporal; TI = temporal inferior; TS = temporal superior.

**Table 4:** Differences in RPC vessel density among patients and controls.

	MS+ON vs. Controls		MS−ON vs. Controls		NMOSD+ON vs. Controls		NMOSD−ON vs. Controls	
RPC (%)	β (SE)	*p*-Value	β (SE)	*p*-Value	β (SE)	*p*-Value	β (SE)	*p*-Value
average	−6.209 (1.042)	<0.001	−3.104 (0.711)	<0.001	−12.172 (3.272)	<0.001	−2.372 (0.739)	0.001
ST	−6.548 (1.576)	<0.001	−2.839 (0.859)	<0.001	−13.862 (4.407)	0.002	−3.713 (0.925)	<0.001
SN	−3.466 (1.458)	0.017	−1.207 (1.116)	0.279	−13.171 (3.701)	<0.001	−0.792 (1.235)	0.521
NS	−3.587 (1.223)	0.003	−0.925 (1.014)	0.361	−10.439 (2.991)	<0.001	−1.537 (1.089)	0.158
NI	−5.668 (1.334)	<0.001	−2.585 (0.967)	0.007	−8.474 (3.227)	0.009	−3.458 (1.587)	0.029
IN	−4.557 (1.392)	0.001	−3.820 (1.050)	<0.001	−13.709 (3.694)	<0.001	−1.661 (1.132)	0.142
IT	−5.824 (1.403)	<0.001	−3.843 (1.001)	<0.001	−16.532 (4.808)	<0.001	−3.724 (1.251)	0.003
TI	−10.603 (1.600)	<0.001	−5.770 (1.031)	<0.001	−11.034 (2.691)	<0.001	−2.390 (1.859)	0.198
TS	−9.955 (1.700)	<0.001	−4.653 (0.939)	<0.001	−12.339 (2.626)	<0.001	−2.413 (1.158)	0.037
	**MS+ON vs. MS−ON**		**NMOSD+ON vs. NMOSD−ON**		**NMOSD+ON vs. MS+ON**		**NMOSD−ON vs. MS−ON**	
**RPC (%)**	**β** **(SE)**	***p*** **-** **Value**	**β** **(SE)**	***p*** **-** **Value**	**β** **(SE)**	***p*** **-** **Value**	**β** **(SE)**	***p*** **-** **Value**
average	−3.106 (0.906)	<0.001	−9.800 (3.266)	0.003	−5.963 (3.364)	0.076	0.731 (0.756)	0.333
ST	−3.709 (1.551)	0.017	−10.148 (4.356)	0.020	−7.313 (4.628)	0.114	−0.874 (1.055)	0.408
SN	−2.259 (1.367)	0.098	−12.380 (3.761)	<0.001	−9.706 (3.854)	0.012	0.415 (1.342)	0.757
NS	−2.662 (1.092)	0.015	−8.902 (2.960)	0.003	−6.851 (3.055)	0.025	−0.611 (1.051)	0.561
NI	−3.083 (1.173)	0.009	−5.016 (3.440)	0.145	−2.806 (3.362)	0.404	−0.873 (1.601)	0.586
IN	−0.738 (1.128)	0.513	−12.047 (3.670)	0.001	−9.151 (3.780)	0.015	2.159 (1.044)	0.039
IT	−1.981 (1.402)	0.158	−12.808 (4.659)	0.006	−10.708 (4.925)	0.030	0.119 (1.317)	0.928
TI	−4.833 (1.444)	<0.001	−8.644 (3.206)	0.007	−0.431 (2.976)	0.885	3.380 (1.890)	0.074
TS	−5.302 (1.508)	<0.001	−9.926 (2.757)	<0.001	−2.384 (2.967)	0.422	2.240 (1.116)	0.045

β = regression coefficient; IN = inferior nasal; IT = inferior temporal; MS = multiple sclerosis; NI = nasal inferior; NMOSD = neuromyelitis optica spectrum disorder; NS = nasal superior; ON = optic neuritis; RPC = radial peripapillary capillaries; SE= standard error; SN = superior nasal; ST = superior temporal; TI = temporal inferior; TS = temporal superior.

## Data Availability

The data presented in this study are available on reasonable request from the corresponding author.

## References

[1] Filippi M., Bar-Or A., Piehl F., Preziosa P., Solari A., Vukusic S., Rocca M.A. (2018). Multiple sclerosis. Nat. Rev. Dis. Primers.

[2] Jasiak-Zatonska M., Kalinowska-Lyszczarz A., Michalak S., Kozubski W. (2016). The immunology of neuromyelitis optica-current knowledge, clinical implications, controversies and future perspectives. Int. J. Mol. Sci..

[3] Jarius S., Wildemann B., Paul F. (2014). Neuromyelitis optica: Clinical features, immunopathogenesis and treatment. Clin. Exp. Immunol..

[4] Kawachi I. (2017). Clinical characteristics of autoimmune optic neuritis. Clin. Exp. Neuroimmunol..

[5] de Seze J. (2013). Inflammatory optic neuritis: From multiple sclerosis to neuromyelitis optica. Neuroophthalmology.

[6] Srikajon J., Siritho S., Ngamsombat C., Prayoonwiwat N., Chirapapaisan N., Siriraj Neuroimmunology Research Group (2018). Differences in clinical features between optic neuritis in neuromyelitis optica spectrum disorders and in multiple sclerosis. Mult. Scler. J. Exp. Transl. Clin..

[7] Hokari M., Yokoseki A., Arakawa M., Saji E., Yanagawa K., Yanagimura F., Toyoshima Y., Okamoto K., Ueki S., Hatase T. (2016). Clinicopathological features in anterior visual pathway in neuromyelitis optica. Ann. Neurol..

[8] Naismith R.T., Tutlam N.T., Xu J., Klawiter E.C., Shepherd J., Trinkaus K., Song S.K., Cross A.H. (2009). Optical coherence tomography differs in neuromyelitis optica compared with multiple sclerosis. Neurology.

[9] Campbell J.P., Zhang M., Hwang T.S., Bailey S.T., Wilson D.J., Jia Y., Huang D. (2017). Detailed vascular anatomy of the human retina by projection-resolved optical coherence tomography angiography. Sci. Rep..

[10] Cennamo G., Carotenuto A., Montorio D., Petracca M., Moccia M., Melenzane A., Tranfa F., Lamberti A., Spiezia A.L., Servillo G. (2020). Peripapillary Vessel Density as Early Biomarker in Multiple Sclerosis. Front. Neurol..

[11] Spain R.I., Liu L., Zhang X., Jia Y., Tan O., Bourdette D., Huang D. (2018). Optical coherence tomography angiography enhances the detection of optic nerve damage in multiple sclerosis. Br. J. Ophthalmol..

[12] Wang X., Jia Y., Spain R., Potsaid B., Liu J.J., Baumann B., Hornegger J., Fujimoto J.G., Wu Q., Huang D. (2014). Optical coherence tomography angiography of optic nerve head and parafovea in multiple sclerosis. Br. J. Ophthalmol..

[13] Huang Y., Zhou L., ZhangBao J., Cai T., Wang B., Li X., Wang L., Lu C., Zhao C., Lu J. (2019). Peripapillary and parafoveal vascular network assessment by optical coherence tomography angiography in aquaporin-4 antibody-positive neuromyelitis optica spectrum disorders. Br. J. Ophthalmol..

[14] Chen Y., Shi C., Zhou L., Huang S., Shen M., He Z. (2020). The detection of retina microvascular density in subclinical aquaporin-4 antibody seropositive neuromyelitis optica spectrum disorders. Front. Neurol..

[15] Thompson A.J., Banwell B.L., Barkhof F., Carroll W.M., Coetzee T., Comi G., Correale J., Fazekas F., Filippi M., Freedman M.S. (2018). Diagnosis of multiple sclerosis: 2017 revisions of the McDonald criteria. Lancet Neurol..

[16] Wingerchuk D.M., Banwell B., Bennett J.L., Cabre P., Carroll W., Chitnis T., de Seze J., Fujihara K., Greenberg B., Jacob A. (2015). International consensus diagnostic criteria for neuromyelitis optica spectrum disorders. Neurology.

[17] Huang D., Jia Y., Gao S.S., Lumbroso B., Rispoli M. (2016). Optical coherence tomography angiography using the Optovue device. Dev. Ophthalmol..

[18] Jia Y., Tan O., Tokayer J., Potsaid B., Wang Y., Liu J.J., Kraus M.F., Subhash H., Fujimoto J.G., Hornegger J. (2012). Split-spectrum amplitude-decorrelation angiography with optical coherence tomography. Opt. Express..

[19] Ferrara D., Waheed N.K., Duker J.S. (2016). Investigating the choriocapillaris and choroidal vasculature with new optical coherence tomography technologies. Prog. Retin. Eye Res..

[20] Green A.J., Cree B.A. (2009). Distinctive retinal nerve fibre layer and vascular changes in neuromyelitis optica following optic neuritis. J. Neurol. Neurosurg. Psychiatry.

[21] Verkman A.S., Ruiz-Ederra J., Levin M.H. (2008). Functions of aquaporins in the eye. Prog. Retin. Eye Res..

[22] Mandler R.N., Davis L.E., Jeffery D.R., Kornfeld M. (1993). Devic’s neuromyelitis optica: A clinicopathological study of 8 patients. Ann. Neurol..

[23] Lucchinetti C.F., Guo Y., Popescu B.F., Fujihara K., Itoyama Y., Misu T. (2014). The pathology of an autoimmune astrocytopathy: Lessons learned from neuromyelitis optica. Brain Pathol..

[24] Outteryck O., Majed B., Defoort-Dhellemmes S., Vermersch P., Zéphir H. (2015). A comparative optical coherence tomography study in neuromyelitis optica spectrum disorder and multiple sclerosis. Mult. Scler..

[25] Nakamura M., Nakazawa T., Doi H., Hariya T., Omodaka K., Misu T., Takahashi T., Fujihara K., Nishida K. (2010). Early high-dose intravenous methylprednisolone is effective in preserving retinal nerve fiber layer thickness in patients with neuromyelitis optica. Graefes Arch. Clin. Exp. Ophthalmol..

[26] FitzGibbon T., Taylor S.F. (2012). Mean retinal ganglion cell axon diameter varies with location in the human retina. Jpn. J. Ophthalmol..

[27] Evangelou N., Konz D., Esiri M.M., Smith S., Palace J., Matthews P.M. (2001). Size-selective neuronal changes in the anterior optic pathways suggest a differential susceptibility to injury in multiple sclerosis. Brain.

[28] Petzold A., de Boer J.F., Schippling S., Vermersch P., Kardon R., Green A., Calabresi P.A., Polman C. (2010). Optical coherence tomography in multiple sclerosis: A systematic review and meta-analysis. Lancet Neurol..

